# Rise of nations: Why do empires expand and fall?

**DOI:** 10.1063/5.0004795

**Published:** 2020-09-01

**Authors:** S. Vakulenko, D. A. Lyakhov, A. G. Weber, D. Lukichev, D. L. Michels

**Affiliations:** 1 Institute of Problems of Mechanical Engineering, Russian Academy of Sciences, Bolshoj Avenue, 61, 199178 St. Petersburg, Russian Federation; 2 Computational Sciences Group, Visual Computing Center, King Abdullah University of Science and Technology, Thuwal 23955-6900, Kingdom of Saudi Arabia; 3 Institute of Computer Science II, University of Bonn, Regina-Pacis-Weg 3, 53113 Bonn, Germany; 4 Faculty of Control Systems and Industrial Robotics School of Computer Technologies and Controls, ITMO University, Kronverkskiy Avenue, 49, 197101 St. Petersburg, Russian Federation; 5 Computer, Electrical and Mathematical Science and Engineering Division, King Abdullah University of Science and Technology, Thuwal 23955-6900, Kingdom of Saudi Arabia

## Abstract

We consider centralized networks composed of multiple satellites arranged around a few dominating super-egoistic centers. These so-called empires are organized using a divide and rule framework enforcing strong center–satellite interactions while keeping the pairwise interactions between the satellites sufficiently weak. We present a stochastic stability analysis, in which we consider these dynamical systems as stable if the centers have sufficient resources while the satellites have no value. Our model is based on a Hopfield type network that proved its significance in the field of artificial intelligence. Using this model, it is shown that the divide and rule framework provides important advantages: it allows for completely controlling the dynamics in a straight-forward way by adjusting center–satellite interactions. Moreover, it is shown that such empires should only have a single ruling center to provide sufficient stability. To survive, empires should have switching mechanisms implementing adequate behavior models by choosing appropriate local attractors in order to correctly respond to internal and external challenges. By an analogy with Bose–Einstein condensation, we show that if the noise correlations are negative for each pair of nodes, then the most stable structure with respect to noise is a globally connected network. For social systems, we show that controllability by their centers is only possible if the centers evolve slowly. Except for short periods when the state approaches a certain stable state, the development of such structures is very slow and negatively correlated with the size of the system’s structure. Hence, increasing size eventually ends up in the “control trap.”

Complex networks appear in many applications in biology, ecology, economics, and social sciences, as well as in artificial intelligence and machine learning. Within the last few decades, the analysis of dynamics and stability of such networks received great attention. A key question asks for the stability of network states under the influence of noise. In this regard, we study a dynamical model describing a network, which is composed of a small number of center nodes and several weakly connected satellite nodes. Centers interact with the satellites according to a “divide and rule” scheme, which implies that satellite-satellite interactions are weak or are totally absent. We refer to such structures as “empires.” For such networks, one can show that their dynamics may be very complex, with a single restriction that the attractor dimension is smaller than the number of centers. However, to provide network state stability under noise, we should increase the number of satellites obtaining a slow down in the empire dynamics. It shows that empires, earlier or later, fall into a control trap: to support the dynamical regime, they should have many satellites, but then their evolution becomes slow. We show that if the noise correlations are negative for each pair of nodes, then the most stable structure of an empire with respect to noise is a globally connected one. In the opposite case, this means that the connectivity of centers in an empire should be bounded.

## I. INTRODUCTION

The introduction of the *divide and rule* concept (also *divide and conquer* or *divide et impera* in its Latin formulation) is partly attributed to the Florentine diplomat and political theorist Niccolò Machiavelli who explained in his 16th-century political treatise *The Prince* (*Il Principe*)^1,2^ to Lorenzo di Piero de’ Medici, at that time the ruler of Florence, how to increase and maintain his power. In fact, this maxim was already practiced in the legal organization of the ancient Roman civilization. The individual member states of the old Roman Empire were only permitted to establish contracts with the central power in Rome. In contrast, contracting with each other was prohibited. In addition, Rome actively enforced a distinct diversity of the individual allies. Hereby, the spectrum of valence ranged from the subjugated ones (*subiecti*), over allies (*foederati* or *socii*) up to legally equated friends of the Roman people (*amici populi Romani*), who were granted Roman citizenship (*civitas Romana*) for their faithfulness. Within this staging, states could empower themselves through good conduct, including varying degrees of self-government. Even the Roman politician and military general Gaius Julius Caesar, who played a critical role in the events that led to the demise of the Roman Republic and the rise of the Roman Empire, already employed the *divide and rule* strategy in order to easily defeat the militarily strong Gauls since Vercingetorix’s attempt to unite the Gauls against Roman invasion came too late.^3^

Since ancient times, this principle seems to be common across different civilizations, cultures, and epochs, partially causing fatal effects until today, for example, as consequences of colonialism, as in the Sykes–Picot agreement between the United Kingdom and France defining their mutually agreed spheres of influence and control in the Middle East.^4^ The contractual partners acted as super-egoistic colonial rulers without taking ethnic and cultural structures appropriately into account in their demarcation agreement. As a consequence of their kleptocratic acting, the colonial rulers were unable to establish a stable order for the peoples living there, for which reason this agreement is today referred to as a major cause of conflicts in the region.^5^

The Rhineland-born German-Italian sociologist Robert Michels, one of the founding fathers of modern political science, introduced in his early 20th-century main work *Political Parties* a sociological study of the oligarchical tendencies of modern democracy,^6,7^ the *iron law of oligarchy* claiming that being ruled by an elite (oligarchy) is inevitable as an “iron law” within any large-scale organization, which is openly committed to democratic principles. Their natural transition into large bureaucracies ruled by only a few is a consequence of “tactical and technical necessities” of the organization caused by the increasing complexity of duties requiring specific skills. The direct involvement of average organization members in general decision-making is severely limited by the growing number and complexity of issues and the large number of members prevents regular contact so that the organization’s leadership can apply the principle of *divide and rule*.^6,7^ Given these general findings, it does not seem surprising that this concept is nowadays also mentioned beyond structures in state politics, as in modern economics as a strategy for market action in order to get the most out of the players in a competitive market or in management studies where *divide and rule* is, among other things, noticed as a common strategy by corporate psychopaths to help consolidate and advance their grip on power in the corporate hierarchy.^8^ Moreover, the *divide and rule* approach is also systematically applied at the management level of corporate companies. In this regard, a recent study specifically analyzes the situation at Walmart Inc., an American multinational retail corporation operating a chain of hypermarkets, discount department stores, and grocery stores.^9^

In order to understand these phenomena from a mathematical point of view, we use network models. The last decades’ studies for topological structures of economical, social, and gene networks have received great attention, for example,^10–13^ among many others. In particular, in Ref. 12, some basic structures are found such as FGR (“fit-get-rich”) and “winner-takes-all” ones. In the first case, the networks have many strongly connected centers; however, those centers share only a small part of all links. The second regime can be described by a beautiful analogy with quantum physics, namely, with Bose–Einstein (BE) condensation, which exhibits a winner-takes-all phenomenon, where the largest connected node also always acquires a finite fraction of links.^12^ This concept found applications in many domains. In particular, in the paper,^14^ the problem of collective decision-making as a second order phase-transition is considered. This problem occurs in heterogeneous information-oriented communities possessing frequent information exchange between individuals. In Ref. 14, the quantum-like model of simplified two-level cognitive systems (TLCS) interacting with a socially important (contextual) information field is proposed, where Bose–Einstein condensation appears.

In this paper, we are going to study not only topological structures and phase transitions, but also dynamical and stochastic properties of networks. To this end, we consider a particular class of networks, studied (in a non-stochastic case) in Refs. 15 and 16, where the name “centralized networks” was established.

Centralized networks are composed of a few center nodes surrounded by a number of satellite nodes. The centers exercise control over the lower-level satellite components directly using a binary power hierarchy realized by the application of a strict *divide and rule* framework allowing for the active supervision of the lower-level components.

Next to their importance with respect to social structures, we would like to mention here that *divide and rule* networks are omnipresent in the natural sciences, for example, as dynamical models for gene regulation and neural networks.^16,17^ In this context, it was shown that such networks can generate complicated (including chaotic) attractors and exhibit different non-trivial bifurcations. Actually, one can show that such networks generate completely structurally stable dynamics defined by systems of an n differential equation in a compact domain where n is the number of centers.

In this paper, a rigorous mathematical model is devised and employed to explain structural changes in large social and economical systems as described above. We study centralized networks organized according to the *divide and rule* principle in its general structure (for brevity, we refer to such networks as empires). Empires consist of only a few of center nodes interacting with a number of satellite nodes. We further assume that the main interactions within such an empire are of a center–center or center–satellite type. In contrast, the pairwise interaction between satellites is either completely absent or at least sufficiently weak. Using a time-continuous description, this model is embedded into a dynamical system described by a set of differential equations. In our paper, this set describes a Hopfield neural network. Retrospectively, this kind of neural networks was discovered by Little in 1974^18^ and then later popularized by Hopfield in 1982.^19^ The Hopfield model has many classical applications in artificial intelligence, gene networks, and spin glasses.^19–24^ There are recent investigations of Hopfield networks in general non-smooth constrained convex optimization^25^ and quantum computing.^26^ The Hopfield model was also applied to social systems in Refs. 27 and 28. The paper^27^ explains why in social systems there appear a polarization in antagonistic groups even in the absence of competition. The work^28^ demonstrates that both the individual’s personality and the connections within a group are important in shaping the entire group dynamics and that the Hopfield neural network can model social groups and group dynamics. Note that earlier in the paper,^29^ the divide and rule principle was applied in another way. Using the Ising model, it is shown in Ref. 29 that in order to force a society to adopt a new point of view, one needs to break its homogeneity.

Our main aim is to study the empire’s stochastic stability of kleptocratic kind for which we consider these systems as stable if the centers have sufficient resources. In contrast, the satellites have no value at all. However, in such a model of a kleptocratic system, even the super-egoistic centers are motivated to support the satellites given the fact that if the satellites are not functioning sufficiently well, then finally the centers lose resources. In other words, in an empire, centers and satellites are coupled in a way that the survival of the satellites is a critical asset from the centers’ perspective. We show that the *divide and rule* framework provides important advantages: it allows for completely controlling the dynamics in a straightforward way by adjusting center–satellite interactions. Periodic (respectively, chaotic) regimes are possible if there are more than two (respectively, three) centers. We show that for a single center, the attractor is always a set of steady states for fixed network parameters. Hence, to avoid chaos, such empires should only have a single ruling center.

We also investigate the stochastic stability of equilibrium states in such centralized networks. Under certain assumptions, it is shown that the probability to be in this state within a time T can be estimated by an interesting relation, which admits an analogy with physics.^12^ It can be connected with energy of some system of particles occupying discrete states. The minimum of this energy corresponds to the maximal probability to stay in the steady state. If the noises acting on nodes are not correlated, then the form of the energy shows that we are dealing with a system of non-interacting particles.

We are seeking for the network’s topology, which corresponds to the maximal robustness with respect to the noise, and in the state of the maximal robustness, the energy E(W) is minimal. Here, we observe a bifurcation induced by noise. Depending on the correlation sign, we have sharply different optimal network topology. If the noise correlations are negative for each pair of nodes, then the corresponding interaction term in the energy is positive, and we obtain that the most stable structure is a globally connected network, where all nodes are connected (the corresponding graph is complete); i.e., a globally connected system is more stable. However, if the noise correlations are positive, then the optimal structure consists of many nodes with identical degrees and the corresponding graph is not complete: its mean connectivity is bounded, even as the number of nodes is large.

This paper is organized as follows. In Sec. II, we precisely describe our mathematical model and introduce required definitions. The systems under investigation are closely related to Hopfield networks.^19^ The stability of all centers and a whole network are investigated in Sec. III. In Sec. IV, we show that the dynamics of the system is completely controlled by the adjustment of center–satellite interactions. In Sec. V, we consider effects induced by a weak interaction between satellites by numerical simulations and asymptotical methods. Final conclusions are given in Sec. VI.

## II. PROBLEM SETUP

A network consists of N nodes with activities $u_{i}$. The nodes can be interpreted as regions, and the viability of these nodes is characterized by the assigned activities.

Many important network models can be represented by the following Hopfield system with continuous time:

$$\frac{du_{i}}{dt}=\sigma \left(\sum_{j=1}^{N}K_{ij}u_{j}-h_{i}-\xi_{i}(t)\right)-\lambda_{i}u_{i}\;,\;i=1,\ldots ,N,$$ (1)

where $u_{i}(t)$ are node activities, K is the N×N interaction matrix, $h_{i}$ are activation thresholds, and σ is a smoothly increasing sigmoidal function such that

$$0\leq \sigma (S)\leq 1,\;\lim_{S\to -\infty }\sigma (S)=0,\;\lim_{S\to +\infty }\sigma (S)=1.$$

We set the initial condition

$$u_{i}(0)=u_{i}^{\left(0\right)}.$$ (2)

The Hopfield model (1) involves parameters $h_{i}$ that can be considered as thresholds for resources accumulated by the ith node. These resources, which define the activity of the nodes, are given by the sums $S_{i}=\sum_{j=1}^{N}K_{ij}u_{j}-h_{i}$, and they are subject to the action of fluctuations $\xi_{i}(t)$.

In this paper, we will apply the general model (1) to describe social structures. In fact, in this model, we take into account the following main features of social and economical structures: (1) they consist of nodes having different levels of activity, (2) the nodes interact and their interaction is defined by a matrix, (3) node activities are saturated, and (4) there exist inertia parameters $\lambda_{i},{\tilde{\lambda }}_{j}$, which define the rate of time evolution for the states.

We suppose that random processes $\xi_{i}(t)$ satisfy the following assumption:

Assumption (random processes are Markov) **M**

*Quantities $\xi_{i}(t)$ are Markov processes with values in* R, *trajectories $\xi_{i}(t)$ are continuous functions of t, and processes $\xi_{i}(t),\xi_{j}(t)$ are independent for i≠j.*

Let us fix continuous trajectories $\xi_{i}(t)$. Then, for t≥0, solutions u(t) of the Cauchy problem defined by Eqs. (1) and (2) uniquely exist because of the boundedness, smoothness of σ, and assumption **M**. Moreover, we obtain *a priori* estimates,

$$0<u_{i}(t)<1/\lambda_{i},\;t>0.$$

### A. *Divide and rule* model for Hopfield networks

In order to obtain our results for systems defined by (1), we do not use any special assumptions regarding the network topology. However, we suppose that there are two types of network components: centers and satellites. In order to take these types of nodes into account, we use a distinct nomenclature $q_{j}$ for centers, j=1,…,n, and $w_{i}$ for the satellites, $i=1,\ldots ,N-n=N_{s}$. The real matrix entry $A_{ji}$ defines the intensity of the action of the satellite node i on the center node j. Similarly, the $n\times N_{s}$ matrix B, the $N_{s}\times N_{s}$ matrix C, and the n×n matrix D define the action of the centers on the satellites, the interactions between the satellites, and the interactions between the centers, respectively. In order to simplify formulas, we make use of the abbreviated notation,

$$\sum_{j=1}^{n}D_{ij}q_{j}={\text{D}}_{i}q,\;\sum_{k=1}^{N}C_{jk}w_{k}={\text{C}}_{j}w$$

so that (1) can be rewritten as follows:

$$\begin{array}{rl} \kappa^{-1}\frac{dq_{j}}{dt} & =\sigma \left({\text{A}}_{j}w+{\text{D}}_{j}q-h_{j}\right)-\lambda_{j}q_{j}, \end{array}$$ (3)

$$\begin{array}{rl} \frac{dw_{i}}{dt} & =\sigma \left({\text{B}}_{i}q+{\text{C}}_{i}w-{\tilde{h}}_{i}+\xi_{i}(t)\right)-{\tilde{\lambda }}_{i}w_{i}, \end{array}$$ (4)

where $i=1,\ldots ,N_{s}$ and j=1,…,n. Here, the unknown functions $w_{i}(t),q_{j}(t)$ are defined for t≥0. We assume that κ is a positive parameter. For small κ, the variables $w_{i}$ are fast with respect to variables $q_{j}$. We set the initial conditions to

$$w_{i}(0)={\tilde{\phi }}_{i}\geq 0,\;q_{j}(0)=\phi_{j}\geq 0.$$ (5)

It is natural to assume that all states are initially non-negative. Moreover, it is clear that they stay non-negative for all times. We suppose, in Eqs. (3) and (4), that noises act on satellites only. The more sophisticated case when noises affect centers is postponed for future works.

**FIG. 1.**
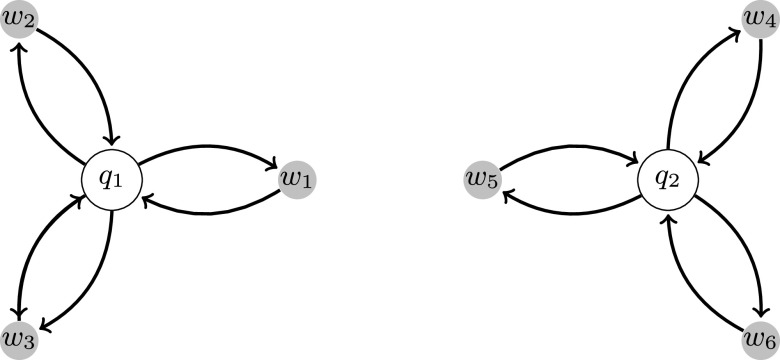
This image shows an example of a *divide and rule* network with n=2 and $N_{s}=6$. The graph consists of eight nodes denoted by $q_{1},q_{2},w_{1},w_{2},w_{3},w_{4},w_{5}$, and $w_{6}$. The set $\left\{q_{1},q_{2}\right\}$ is the set of centers C. Let $S(q_{j})$ denote the set of satellites connected with the center $q_{j}$, then, for example, $S^{*}(q_{1})=\{w_{1},w_{2},w_{3}\}$ and $S^{*}(q_{2})=\{w_{4},w_{5},w_{6}\}$.

The *divide and rule* model is a particular case of Eq. (1) for small entries $C_{ij}$, when the satellite–satellite interactions are weak. In this case, the structure of the interactions can be illustrated by Fig. 1.

### B. Viability problems for networks under stochastic impact

We consider the viability problems for systems (3) and (4). We suppose that the system is viable if the activities of the centers are sufficiently large. The viability domain is defined as a set $\Pi =\{q\in {\text{R}}_{+}^{m}:q_{i}\geq \delta_{i}\;\forall i\}$, where $\delta_{i}>0$ are some thresholds corresponding to a minimal possible activity.

The general approach to viability problems was initially developed by Jean-Pierre Aubin.^30^

As a measure of the stochastic stability of the system within an interval [0,T] with the initial state $u^{0}=(q^{0},w^{0})$, we consider the probability that q(t)∈Π,

$$P(P,\Pi ,u^{0},T_{1},T_{2})=\mathrm{Prob}\{q(t)\in \Pi \;\text{for each}\;t\in [T_{1},T_{2}]\},$$ (6)

where $q=(q_{1},\ldots ,q_{m})$. This probability depends on the system parameters P and the stability domain Π. It defines the system viability on the time interval $\left[T_{1},T_{2}\right]$.

### C. Hopfield network viability under random fluctuations

For the Hopfield networks defined by (1), we assume that the viability domain $\Pi \subset {\text{R}}^{N}$ is a subset of the nonnegative cone ${\text{R}}_{+}^{N}=\{u_{i}\geq 0\}$. Moreover, if Π is a subset of the cone,

$${\text{Con}}_{i_{1},\ldots ,i_{s}}=\{u:u\in {\text{R}}_{+}^{N},\;u_{i_{l}}>0,\;l=1,\ldots ,s\}$$ (7)

for the maximal possible s. Then, we say that $\{i_{1},\ldots ,i_{s}\}=K$ is the *set of key indices* and $N_{key}$ is its cardinality.

Remark 1 If i is a key index, then we assume $u_{i}$ is positive (ith node corresponds to the active one). The key index corresponds to the important administrative center of the empire. This approach is also rather fruitful in biology, where such nodes correspond to genes important for the organism’s functionality. In fact, it is well known (see the seminal paper^31^) that important genes are hubs in gene networks.

### D. Parameters of Hopfield networks

Each interaction matrix K generates a directed graph with N nodes and at most N(N−1)/2 edges. We assume that ith and jth nodes are connected by a directed edge if the corresponding entry $K_{ij}\neq 0$. Biological circuits are usually far away from being completely connected.^10^ Valency (degree, connectivity) of a node (vertex) is the number of the edges, which enter this node,

$$V_{i}=\text{Card}(E_{i})=|E_{i}|,\;E_{i}=\{j:K_{ij}\neq 0\}.$$

For each fixed node i, we have a valency $V_{i}\leq N$: only the $V_{i}$ among the entries $K_{ij}$ is not equal to zero. For quantitative measurements, we will use the minimal connectivity of the key nodes defined by the parameter

$$V=\min_{i\in K}V_{i},$$

where the minimum is taken over by all key indices $i_{1},\ldots ,i_{s}\in K$. The number of such indices is denoted as $N_{key}$.

We estimate the stochastic stability via V and the following parameters:

$$K_{*}=\max_{i,j}{\left(K_{ij}\right)}_{+},\;R=\max_{i}\lambda_{i}^{-1},$$ (8)

where $f_{+}=f$ for f>0 and $f_{+}=0$ for f≤0.

## III. STABILITY OF ALL CENTERS AND THE WHOLE NETWORK

In this section, we consider the probability $P_{\mathrm{sur},\mathrm{cent}}$ that all centers are viable in the time interval [0,T] and the probability $P_{\mathrm{sur},\mathrm{net}}$ that all nodes are viable. Our aim is to understand which network topology corresponds to the maximal viability probability. The first variant describes a kleptocratic regime, when we ignore satellite viability. In a kleptocratic system of course, the main problem is to conserve centers.^32^

The second case, when we must obtain the maximum of $P_{\mathrm{sur},\mathrm{net}}$, can be interpreted as a “democratic regime.” We fix the total number of nodes in the network N, but for the kleptocratic regime, we vary the number of the centers n to study how the stability depends on the center number. We will consider the case of arbitrary network topology and correlated noises: there are possible correlations between noises acting on different nodes.

Note that in reality, existing free-scale networks distinguishing hubs (centers) and satellites are not quite a trivial problem; therefore, our model of kleptocratic system is an idealization.

To simplify our analysis, we consider the Gaussian noises and the following classical time discrete variant of the Hopfield equations (1),

$$u_{i}(t+1)=\sigma (\sum_{j}K_{ij}u_{j}(t)-h_{i}-\xi_{i}(t)),$$ (9)

where $\xi_{i}(t)$, i=1,2,…,N are time discrete Gaussian random processes simulating a discrete white noise in t. For each t, we sample real valued $\xi_{i}(t)$ by the multivariate Gaussian density with the zero mean,

$$\rho_{G}(\xi )={\left(2\pi \right)}^{-N/2}|\det C{|}^{-1/2}\exp\left(-\frac{1}{2}\xi^{tr}C^{-1}\xi \right),$$ (10)

where C is a positively definite symmetric covariance matrix.

Model (9) is the paradigm of the neural network and spin glass theory. It is well known that in the absence of noises, this system can simulate all Turing machines^33^ and generate all structurally stable dynamics.^15,16^ To avoid considerations of complicated dynamical regimes, we make additional assumptions; namely, let σ be the Heaviside step function [then $u_{i}(t)\in \{0,1\}$], and let $u=(1,1,\ldots ,1)=u_{eq}$ be a steady state of (9). We introduce the probabilities $P_{\mathrm{sur},\mathrm{cent}}$ and $P_{\mathrm{sur},\mathrm{net}}$ by

$$P_{\mathrm{sur},\mathrm{cent}}=\mathrm{Prob}\{u_{i}(t+1)=1\forall i\in K|u_{i}(t)=1\forall i\},$$ (11)

where K is the set of i corresponding to centers, and

$$P_{\mathrm{sur},\mathrm{net}}=\mathrm{Prob}\{u_{i}(t+1)=1\forall i|u_{i}(t)=1\forall i\},$$ (12)

respectively. Those probabilities serve as measures of stochastical stability of an active network steady state under noises. In that steady state, all nodes are active. Note that if the threshold $h_{i}$ is negative and $|h_{i}|>>1$ for all satellite indices i, then $P_{\mathrm{sur},\mathrm{net}}\approx P_{\mathrm{sur},\mathrm{cent}}$. In this case, the satellites can be considered as an external field for centers since the fluctuations weakly affect their states.

To compute the probabilities $P_{\mathrm{sur},\mathrm{cent}}$ and $P_{\mathrm{sur},\mathrm{net}}$, we introduce the quantities analogous to the connectivity $V_{i}$, which, however, take into account the interaction magnitudes between the nodes and thresholds,

$$W_{i}=-h_{i}+\sum_{j}K_{ij}.$$ (13)

The quantity $W_{i}$ can be interpreted as a force acting on the node i, which is induced by all other nodes. If all $K_{ij}$ are equal, $K_{ij}=K$ and $h_{i}=h$, then $W_{i}$ is, up to constant, the degree (connectivity) of the node i. We can express our probabilities via W as follows:

$$P_{\mathrm{sur},\mathrm{net}}=\int_{\Omega_{\mathrm{net}}(W)}\rho_{G}(\xi )d^{N}\xi$$ (14)

and

$$P_{\mathrm{sur},\mathrm{cent}}=\int_{\Omega_{\mathrm{cent}}(W)}\rho_{G}(\xi )d^{N}\xi ,$$ (15)

where the integration domains $\Omega_{\mathrm{net}}$ and $\Omega_{\mathrm{cent}}$ are defined by

$$\Omega_{\mathrm{net}}=\{\xi_{i}<W_{i}\;\forall i\},\;\Omega_{\mathrm{cent}}=\{\xi_{i}<W_{i}\;\forall i\in K\}.$$

### A. Independent noises

If the fluctuations $\xi_{i}$ are independent, then the matrix C is diagonal. Let $C^{-1}=\mathrm{diag}(\beta ,\ldots ,\beta )$, where β>0. Then, we obtain

$$P_{\mathrm{sur},\mathrm{cent}}=\exp(-E_{0}(W)),$$ (16)

where $E_{0}(W)$ is

$$E_{0}(W)=-\sum_{i\in K}\ln(1-\exp(-U(W_{i}))),$$ (17)

and where for large $|W_{i}|>>\beta^{-1/2}$,

$$U(W_{i})=\frac{\beta W_{i}^{2}}{2}+\ln|W_{i}|+\frac{1}{2}\ln\beta -\frac{\ln(2\pi )}{2}+O(\beta^{-1}W_{i}^{-2}).$$ (18)

Therefore, for large $W_{i}$, one has

$$E_{0}(W)\approx \sum_{i\in K}\exp(-U(W_{i})).$$ (19)

Analogous relations hold for n=N (the democratic case). We can consider $E_{0}(W)$ as an energy of a system of non-interacting particles. When that energy is minimal, our probability $P_{\mathrm{sur},\mathrm{cent}}$ is maximal; therefore, the optimal network structure maximally robust with respect to the noise can be found by minimization of $E_{0}(W)$.

In the general case of arbitrary $K_{ij}$ the problem is complicated. However, under conditions

$$|K_{ij}|<K_{max},\;h_{i}=h$$ (20)

and large $W_{i}$, we find that for fixed N and in the democratic case (we seek for maximal $P_{\mathrm{sur},\mathrm{net}}$), the minimum of $E_{0}(W)$ attains when we are dealing with a maximally connected network with positive interactions $K_{ij}>0$.

The problem can be simplified under conditions

$$K_{ij}=K>0,\;h_{i}=0.$$ (21)

Let n be the number of the centers and let us denote by π(k) the probability that a center has k connections. Then, under conditions β>>1 and $\beta^{-1/2+\delta }<<W_{i}<<\beta^{p}$, where δ>0 and p>0, we can obtain an asymptotics of $U(W_{i})$ by (18) as follows. We remark that the first term on the right-hand side of (18) is much more than $\beta^{2\delta }$, the second and third terms are order of ln⁡β, the fourth term is O(1), and the last one vanishes at all as β→+∞. Therefore, for large β,

$$U(W_{i})\approx \frac{\beta W_{i}^{2}}{2}(1+O(1)).$$ (22)

Now, the energy $E_{0}(W)$ can be expressed via π(k) as follows:

$$E_{0}\approx E_{0}[\pi ]\approx -\sum_{k_{min}}^{k_{max}}\pi (k)\ln(1-\exp(-\beta K^{2}k^{2}/2)).$$

The expression $E_{0}[\pi ]$ can be interpreted as an energy of a system of non-interacting particles, which can occupy the levels $k=k_{min},\ldots ,k_{max}$ with the inverse temperature $\beta K^{2}$. If the energy E[π] has a minimum, the network system has the maximal robustness. Note that the energy level k is discrete and this ideal gas of “quasiquantum” particles exhibits interesting properties. Namely, the optimal state is similar to a Bose–Einstein condensate when the distribution $\pi (k)=\delta (k-k_{max})$ is localized at $k_{max}$. It corresponds to an “empire” with a single center, where a single node shares an essential part of links. Therefore, the kleptocratic case structure with a single center is more stable.

Let us consider now the problem of minimization $E_{0}(W)$ under condition (20) and the following restriction on the average connectivity $\bar{C}$ of the network:

$$N^{-1}\sum_{i=1}^{N}W_{i}=K\bar{C}.$$ (23)

Then, in the democratic case, the situation changes. To see it, consider the expression (16), where the set K contains all i=1,…,N. We are seeking for the maximum of the right-hand side of (16) with respect to $W_{i}$ under restrictions $W_{i}\in [0,KN]$ and (23). Using the Lagrange multiplier $\lambda_{L}$, we obtain that for the optimal state,

$$\exp(-\beta W_{k}^{2}/2)(\beta W_{k}+W_{k}^{-1})=c(\beta )\lambda_{L}$$ (24)

for each k and where c(β) is a positive constant. We see that all $W_{i}$ are equal. In that network, there are no strong centers.

### B. Strongly correlated noises

The case of the maximally strong correlation arises if $\xi_{i}=c_{i}\xi$, where $c_{i}$ are constants and ξ(t) is a random process with the discrete time taking the values in R. Then, for a given t,

$$P_{\mathrm{sur},\mathrm{net}}=\mathrm{Prob}\{\xi (t)<W_{\text{\{min\}}}\},$$

where $W_{\text{\{min\}}}=c_{i}^{-1}\min W_{i}$. Therefore, to obtain the maximum of $P_{\mathrm{sur},\mathrm{net}}$ we have to resolve a minimax problem: to find $W_{i}$ such that $\min W_{i}=\max$. It is clear that for the fixed number N and a fixed average connectivity $\bar{W}$, the solution is $W_{i}c_{i}^{-1}=\mathrm{const}$; i.e., all nodes are connected similarly (if $c_{i}$ has the same order). We obtain the same result as in the kleptocratic case.

### C. Weakly correlated noises

It is difficult to compute $P_{\mathrm{sur},\mathrm{net}}$ in the case of general Gaussian noises. To handle the problem, we consider the case of a weak correlation, where $C^{-1}=\mathrm{diag}(\beta ,\beta ,\ldots ,\beta )+\tilde{C}$, where $\tilde{C}$ is a small perturbation. Note that then for the covariance matrix C, one has

$$C=\beta^{-1}I-\beta^{-2}\tilde{C}+O(||\tilde{C}|{|}^{3}),$$

where I stands for the identity matrix. The case where all entries of the matrix $\tilde{C}$ are positive (negative), we will refer as cases of the negative (positive) noise correlation. Taking into account only the main terms and correction terms of the first order in $\tilde{C}$, we obtain

$$\ln P_{\mathrm{sur},\mathrm{net}}\approx -E(W)=-(E_{0}(W)+E_{1}(W)),$$

where $E_{0}(W)$ is defined by (19) and

$$E_{1}(W)=-\frac{1}{2}\sum \sum_{i,j}{\tilde{C}}_{ij}\ln\left(1-\exp\left(-\beta \frac{\left(W_{i}^{2}+W_{j}^{2}\right)}{2}\right)\right),$$

is an energy of ”interaction,” induced by noise correlations.

Thus, we obtain that finding an optimal structure minimizing noise impact on the steady state is equivalent to the following standard problem of statistical mechanics: to find the main state of the system with the energy $E(W)=E_{0}(W)+E_{1}(W)$, which corresponds to a system of N interacting particles. We consider that problem under simplifying assumptions (21). In the kleptocratic case, we obtain the same expression, but the sums are taken over all i,j∈K and we have n particles. Note that the values $W_{i}$ are discrete; thus, we are dealing with particles, which have certain quantum properties, and their states lie in a discrete set. Note, moreover, that systems of interacting quantum particles with Bose statistics can exhibit the Bose–Einstein condensation, as it was established by Bogolubov.^34^

The solution of our optimization problem can be found in the two simple cases: ${\tilde{C}}_{ij}=b/N>0$ and ${\tilde{C}}_{ij}=-b/N<0$, where b is a small positive parameter. In terms of noises, depending on the sign of b, we have correlation and anticorrelation: the case b>0 corresponds to negative correlations, and for b<0, we have positive correlations between noises acting on different nodes. To see it, we further simplify the expression for $E_{i}(W)$ supposing β<<1. Then, by removing $\ln W_{i}$ in the relations for $E_{i}(W)$, one obtains

$$E(W)\approx R(W)+\frac{b}{2}R{\left(W\right)}^{2},$$

where

$$R(W)=\sum_{i=1}^{N}\exp(-\beta W_{i}^{2}/2)>0.$$

If b≥0, then the global minimum of the polynomial $R+bR^{2}/2$ attains at R=0; thus, the optimal state of the networks attains at maximal possible center connectivity $W_{i}$. In particular, the structure with a single center sharing the maximum of possible links is most stable.

If b<0, then the minimum of $R+bR^{2}/2$ attains at R=b. Then, for each n, the optimal structure of the networks is absolutely another. Here, the connectivity of each center is bounded and depends on b.

For the democratic case (i=1,…N), we obtain that globally connected networks with the complete graph of interactions are most stable for b>0, but for b<0, the connectivity of this graph should be bounded. We see that the cases b>0 and b<0 are sharply different; therefore, b=0 is a critical value for a transition induced by noise. The result essentially depends on the sign of b, and at b=0, we observe a bifurcation.

A more accurate analysis leads to the same conclusions. We can differ two situations: (A) without restrictions on the average connectivity $\bar{C}$ and (B) with restriction (23) on $\bar{C}$. Consider a more general case (B). Using the Lagrange multiplier, we have relations, which generalize (24),

$$\exp(-\beta W_{k}^{2}/2)(\beta W_{k}+W_{k}^{-1})+b\beta RW_{k}=c(\beta )\lambda_{L}.$$ (25)

In the case b>0, those equations have no solutions. In the case of b<0, we resolve these equations for a given R that gives us $W_{i}=w(R)$, and then, we find R and $\lambda_{L}$ by the conditions

$$N\exp(-\beta w{\left(R\right)}^{2}/2)=R,\;w(R)=\bar{C}.$$

This procedure permits us to find solutions in the interior of the domain $W_{i}\in (0,KN),\;\mathrm{for}\;\mathrm{all}\;i$. Moreover, there are possible solutions corresponding the case when $W_{i}=0$ for some i. One can show that for small b<0, the global minimum corresponds to the studied case where all $W_{i}>0$.

## IV. NETWORK DYNAMICS, TOTAL CONTROL, AND MAXIMALLY FLEXIBLE SYSTEMS

In this section, we state a mathematical approach to describe systems with a total control, where a few of centers would like to completely prescribe the behavior of the whole system. We consider an ideal situation, where we remove the random processes $\xi_{i}$ and thus all random effects. Particularly, we set $\xi_{i}(t)\equiv 0$ for all i in Eq. (1) and in Eqs. (3) and (4). The random effects will be studied numerically in Sec. V C.

How can we describe mathematically systems of total control, which were an ideal for many dictators in the past and wonderfully still stay such an ideal in the present? What are the capabilities of this system and what are its limitations?

To this end, we make use of the concept of inertial manifolds developed to describe the dynamics of infinite dimensional dissipative systems.^35^ An invariant manifold is called inertial if it is a globally attracting set. Such an approach is chosen because, for large times, dynamics of whole big system (possibly, even an infinite dimensional one) with an inertial manifold is determined by a few variables, i.e.,

$$x(t)=g(q)+\tilde{x}(t),$$

where x(t) is the state of the system, q are control variables, and dimx=N>>n=dimq; the function $\tilde{x}$ is exponentially decreasing in t corrections. The dynamics of q is defined by a few dimensional system dq/dt=Q(q). The inertial dynamics usually appears when a big system can be decomposed in slow components and fast ones. Then, we can summarize the inertial dynamics principle simply: for large times, dynamics of a whole system is captured by dynamics of slow components. In such a system, we observe the following dynamical picture: first, we see a fast dynamics within a relatively short time period, when the system state approaches to the inertial manifold and then a slow time evolution of that state on that manifold. The first stage corresponds to an approaching to inertial manifold when the term $\tilde{x}(t)$ is not small yet and the second stage is a motion on the inertial manifold. Inertial dynamics enjoys remarkable stability properties. Under certain assumptions, it is stable under perturbations including stochastic ones, and if an external shock is happened, the effect of this shock is only temporary.

In this section, we first state two theorems obtained in Refs. 16, 17, and 36, and we discuss their consequences for social systems. Those theorems show the occurrence of the inertial dynamics when centers are slow and that the network’s behavior can realize all finite dimensional structurally stable dynamics. Therefore, roughly speaking, all robust dynamics (stable under small perturbations) can be generated by the systems, which satisfy the above formulated properties. Such systems are called as *maximally flexible*.^36^ Also, the concept of maximal flexibility covers the case of chaotic dynamics. In order to show it, let us consider systems of differential equations defined by

dq/dt=Q(q), (26)

(26), which satisfy the following condition:

**Condition structural stability (SS)**. *System (26) generates a global semiflow $S^{t}$, t>0, defined on the n-dimensional closed ball $B^{n}\subset R^{n}$ and having structurally stable (for example, hyperbolic) local attractors $A_{l}$, l=1,…,k*.

Structural stability is a fundamental property of dynamics, which means that the topological structure of the trajectories of system (26) on $A_{l}$ is unaffected by $C^{1}$-small perturbations of the vector field Q. In particular, under small perturbations, hyperbolic rest points remain so and only slightly shift, they cannot be transformed into cycles and vice versa, and hyperbolic cycles cannot become points.

The structurally stable systems can exhibit complicated dynamics, for example, *chaotic* (see Ref. 37 for details).

In the theory of dynamical systems, there are two outstanding results: first, Anosov and Smale proved that there exist structurally stable systems with a chaotic behavior, and furthermore, it was shown by Smale that structurally stable systems, in a sense, are rare (formally, they do not form an open set in the space of all systems for dimensions n>2).^37^

The first theorem shows that total control is possible for *divide and rule* networks when the center’s dynamics is slow.

Theorem 1 **(Center control for *divide and rule* networks)**

Assume that κ>0 is sufficiently small, $\kappa <\kappa_{0}(N),$ and $\lambda_{0}>0$ is sufficiently large. Then, the global semiflow defined by the system (3) and (4) has a $C^{r}$-smooth inertial manifold (where r>1) of the form w=W(q). This implies, in particular, that for large times t, the satellite dynamics is captured completely by the center dynamics,

$$w_{i}(t)=W_{i}(q(t))+{\tilde{W}}_{i}(t),$$ (27)

where the functions $W_{i}$ have asymptotics,

$$W_{i}(q)=\lambda_{i}^{-1}g_{i}(q)+O(\lambda_{0}^{-2})+O(\kappa )$$

and

$$|{\tilde{W}}_{i}(t)|<C_{0}\exp(-\lambda_{0}t),$$

where a positive constant $C_{0}$ depends on initial conditions but uniform in $\lambda_{0}$ and κ and $g_{i}$ are certain smooth functions.

Functions $g_{i}$ have a complicated form, and they can be found in Ref. 17.

Is this theorem applicable to social systems? We think so and illustrate it using the following example.

Following Karl Marx, we assume that Russia turned off the European path of development under Ivan Kalita (1322–1340), when Moscow became the center of the unification of Russian lands. Unification needed a strong state. Note that, according to the inertial dynamics theory, such strong systems should be very stable, and we see that this really is true: after the revolution and civil war, Russia is reborn under the name of the USSR and Stalin turns it into a kind of empire. Russia also endured fantastic chaos during the Time of Troubles (the period in the history of Russia from 1598 to 1613, marked by natural disasters, civil war, the Russian-Polish and Russian-Swedish wars, the most severe state-political and socio-economic crisis), but the Moscow system has revived again. The contemporary Russian system, we think, is also a partial restoration of a tight system of control over the regions after the chaos of the Yeltsin period. Why does this seem to be effective and is it really effective? According to inertial dynamics principles, the fast evolution may be only short (see above). Notice that the fast growth without great sacrifices was in Russia in the period 1880–1913 as a result of Emperor Alexander II serfdom abolishing in the emancipation reform of 1861 when the slaving system of the previous Emperor, Nikolai, I, is weakened.

Theorem 1 is demonstrated in Ref. 17 for the *divide and rule* Hopfield model (3) and (4). Note that the critical value $\kappa_{0}$ decreases as the satellite number $N_{s}$ increases.

Furthermore, *divide and rule* networks satisfy the maximal switchability principle, which was proven for the Hopfield networks.^17^ Let us formulate certain conditions (see Ref. 17).

Let us assume that the system parameters $\text{P}=\{\text{A},\text{B},\text{C},\text{D},h,\tilde{h},\tilde{\lambda },\lambda \}$ satisfy the following conditions:

$$\begin{array}{c} A=\kappa^{-1}\bar{A}, \end{array}$$ (28)

$$\begin{array}{c} |\bar{A}|,|B|,|C|,|D|<c_{0}, \end{array}$$ (29)

$$\begin{array}{c} 0<c_{1}<{\bar{\lambda }}_{i}<c_{2},\;0<{\tilde{\lambda }}_{i}<c_{3}. \end{array}$$ (30)

Here, all positive constants $c_{k}$ are independent of κ for small κ. The smaller the value of κ, the slower the centers’ dynamics are in comparison with its satellites and the center control on satellites is weaker. Serfdom abolishing allowed former serfs quit agriculture moving to cities. For example, the creator of the famous brand Pyotr Arsenievich Smirnov became one of the first peasants who rapidly developed after the abolition of serfdom.

The scaling assumption on A is needed because, as we will prove later, w=O(κ) for small κ. For the same reasons, ${\text{C}}_{i}w$ can be neglected with respect to ${\text{B}}_{i}v$ for small κ, meaning that the action of centers on satellites is dominant with respect to satellites’ mutual interactions. In other words, these conditions describe a divide and rule control principle.

Theorem 2 **(Maximal flexibility theorem for *divide and rule* networks)**

Assume that the dynamical system defined by (26) satisfies conditions (3) and (4). Then, for sufficiently large satellite numbers $N_{s}$, there exist matrices A,B,D and parameters $h,\tilde{h},\lambda ,\tilde{\lambda }$, and κ such that the dynamical system defined by (3) and (4) has local attractors $B_{l}$ topologically equivalent to $A_{l}$. The restrictions of the semiflow $S_{H}^{t}$ to $B_{l}$ are orbitally topologically equivalent to the restrictions of semiflows $S^{t}$ to $A_{l}.$

The proof is based on a classical center manifold technique and on the well known idea that any n-dimensional dynamics can bifurcate from an equilibrium with n-zero eigenvalues if the number of bifurcation parameters is large enough. Such approach was used for finite dimensional systems (see Ref. 38) and after for reaction–diffusion equations^39^ and reaction–diffusion systems.^40^ Let us outline briefly the method.

Let us consider a system of ODEs involving a parameter P. Assume that for each value of P, the system generates a global semiflow $S^{t}$. We obtain then a family F of global semiflows $S_{P}^{t}$, where each semiflow depends on the parameter P. Suppose that for an integer n>0, there is an appropriate value $P_{n}$ of the parameter P such that the corresponding global semiflow $S_{P_{n}}^{t}$ has an n-dimensional finite $C^{1}$-smooth locally invariant manifold $M_{n}$. The semiflow $S_{P_{n}}^{t}$, restricted to $M_{n}$, is defined by a vector field Q on $M^{n}$. Then, we say that the family $S_{P}^{t}$ realizes the vector field Q. The main idea of the proof is that the Hopfield neural networks are capable to realize (within arbitrary small accuracy) all fields.

In our case of the Hopfield model, the parameters P are center–satellite interactions; i.e., control can be performed in a straight-forward way by adjusting center–satellite interactions. In other words, one can say that by tuning those interactions, the reduced dynamics on the inertial manifold can be specified within an arbitrarily small error.

Corollary *Divide and rule* networks are maximally flexible in the sense of the previous comment; in particular, they are capable to generate different kinds of chaotic and periodic dynamical regimes. In particular, any kind of behavior from the chaotic hyperbolic sets can occur in the dynamics of the maximally flexible systems, for example, Anosov flows, Ruelle–Takens–Newhouse chaos,^37^ or Smale’s horseshoes.

In connection with that corollary, it is interesting to discuss which regimes could appear in real dynamics of social and economic systems. In real applications, systems of ODEs seldom enjoy the structural stability property except for dimensions 1 and 2 that is natural in connection with the Smale theorem mentioned above. Therefore, if we are dealing with a two center case, we can expect the appearance of periodical cycles. Typically, in dissipative systems, such cycles are structurally stable. A huge literature is devoted to business cycles, many famous economists (for example, Kuznets, Kondratiev, and Schumpeter, among many others) believed their existence. Modern economic theory declines toward the study of economic fluctuations rather than cycles (see Ref. 41 for an overview).

Note that coexistence of many cycles is a feature of chaos; for example, a chaotic hyperbolic set contains infinitely many unstable cycles.

Theorem 2 shows, moreover, that the systems (3) and (4) can exhibit multistationarity, i.e., they can switch between different local attractors by a choice of an initial state. However, of course, to realize these switches, the network should have a sufficiently large satellite number.

Therefore, we see that divide and rule networks permit to realize the complete control; however, the price of that control is very high: center dynamics should be slow. The larger the empire, the slower it develops.

## V. SINGLE CENTER ASYMPTOTICS AND NUMERICAL SIMULATIONS

In this section, using our model, we study numerically how satellite interactions influence the viability of growing empires *when the number of satellites increases*. In order to do so, we consider the system (1) in the case of a single center, i.e., n=1, and under some simplifications. It takes the form

$$\begin{array}{rl} \kappa^{-1}\frac{dq}{dt} & =\sigma \left(\sum_{j=1}^{N}a_{j}w_{j}-h\right)-q, \end{array}$$ (31)

$$\begin{array}{rl} \frac{dw_{i}}{dt} & =\sigma \left(b_{i}q+\sum_{j=1}^{N}C_{ij}w_{j}-{\tilde{h}}_{i}+\xi_{i}(t)\right)-w_{i}, \end{array}$$ (32)

where i=1,…,N. We set the initial conditions

$$w_{i}(0)\in (0,1),\;q(0)\in (0,1).$$ (33)

In simulations, σ is defined by $\sigma (z)={\left(1+\exp(-z)\right)}^{-1}.$ The parameter κ>0 may be arbitrary, but in order to control the satellites by the center, κ is considered to be sufficiently small. Then, the variables $w_{i}$ are fast with respect to q or, in other words, the parameter $\kappa^{-1}$ represents satellite mobility with respect to the center one. In the analysis for large N, we will use normalized quantity, which is given by the formula $\bar{h}=h/N$.

### A. Asymptotics

We start with the first case, when there are no satellite interactions ($C_{ij}=0$). Numerical simulations show then that all trajectories defined by Eqs. (31) and (32) are convergent; i.e., they tend to equilibrium. For small κ>0, it is a consequence of the existence of a one-dimensional inertial manifold. The dynamics on this manifold is defined by a differential equation dq/dt=W(q), where W is a smooth function such that W<0 for large |q|. For such an equation, all trajectories are converging to $q_{\mathrm{cent}}$, where $q_{\mathrm{cent}}$ is the solution of the equation $W(q_{\mathrm{cent}})=0$.

Then, the equilibrium condition implies

$$\begin{array}{rl} {\bar{w}}_{i}(q_{\mathrm{cent}}) & =\kappa \sigma \left(b_{i}q_{\mathrm{cent}}-{\tilde{h}}_{i}\right), \end{array}$$ (34)

$$\begin{array}{rl} q_{\mathrm{cent}} & =V(q_{\mathrm{cent}})=\sigma \left(N(S(q_{\mathrm{cent}})-\bar{h})\right), \end{array}$$ (35)

where

$$S(q)=N^{-1}\sum_{j=1}^{N}a_{j}{\bar{w}}_{j}(q).$$

Consider the asymptotics of this solution for large N. In the generic case of a parameter choice, we may suppose that the function S(q) has the order 1.^42^ Then, we obtain a piecewise linear network model, which is well studied in many publications.^43^

The set

$$D=\{q\in [0,1]:\;S(q)>\bar{h}\}$$

plays an important role in the asymptotics analysis. It is an interval $\left[d_{1},d_{2}\right]$ or a union of intervals. In the simplest case, when D is an interval, the following situations occur.

(**I**) The interval D contains 1.

(**II**) The interval D contains 0 but does not contain 1.

(**III**) The interval D does not contain both 0 and 1.

In case **I**, we obtain that $q_{\mathrm{cent}}=1$ is a global attractor of the system (31) and (32), if D=[0,1], and $q_{\mathrm{cent}}=1$ is a local attractor if 0∉D. In the second case, we observe bistability with a saddle point $v=d_{1}$. For **II**, we obtain a stable globally attracting equilibrium $v_{\mathrm{cent}}$, $0<v_{\mathrm{cent}}<1$, and in case **III**, we obtain a local equilibrium 0, a saddle point, and a second local attractor within (0,1). If D is a union of disjoint subintervals, then we have multiple equilibria and saddle points.

### B. Perturbation theory for small satellite interactions

Weak random satellite interactions may affect equilibrium states and the center activity depending on three distinct cases: **SM**, satellite mutualism, where the expected values of $C_{ij}>0$; **SC**, satellite competition for center resources, where $EC_{ij}<0$; and the neutral case **SN**, $EC_{ij}=0$. The equilibrium is governed by equations

$$\begin{array}{rl} w_{i}(q_{\mathrm{cent}}) & =\kappa \sigma \left(b_{i}q_{\mathrm{cent}}+\sum_{j=1}^{N}C_{ij}w_{j}-{\tilde{h}}_{i}\right), \end{array}$$ (36)

$$\begin{array}{rl} q_{\mathrm{cent}} & =V(q_{\mathrm{cent}})=\sigma \left(N\left(N^{-1}\sum_{j=1}^{N}a_{j}w_{j}(q_{\mathrm{cent}})-\bar{h}\right)\right). \end{array}$$ (37)

We set $w_{i}={\bar{w}}_{i}+{\tilde{w}}_{i}$, where ${\bar{w}}_{i}$ is defined by Eqs. (34) and (35) and ${\tilde{w}}_{ij}$ is perturbations. For a given function q as a solution of non-perturbed equations, by taking into account smallness of $C_{ij}$, one obtains

$${\tilde{w}}_{i}(q)=\kappa (S_{1,i}+S_{2,i}+\cdots ),$$ (38)

where

$$\begin{array}{rl} S_{1,i} & =\sigma^{\prime }\left(b_{i}q-{\tilde{h}}_{i}\right)\sum_{j=1}^{N}C_{ij}{\bar{w}}_{j},\\ S_{2,i} & =\frac{1}{2}\sigma^{{}^{\prime \prime }}\left(b_{i}q-{\tilde{h}}_{i}\right){\left(\sum_{j=1}^{N}C_{ij}{\bar{w}}_{j}\right)}^{2}. \end{array}$$

In the case **SM** and **SC**, the main contribution in the perturbation ${\tilde{w}}_{i}$ is given by the term $S_{1,i}$.

Consider the case **SM**. Then, $E{\tilde{w}}_{i}(q)>0$; therefore, mutualism leads to an enhancement of satellite activities, and furthermore, the center activity also increases. In contrast, in the case **SC**, we have an opposite picture: $E{\tilde{w}}_{i}(q)<0$, which implies the fall of the satellite activities, and as a consequence, the center activity also decreases.

**FIG. 2.**
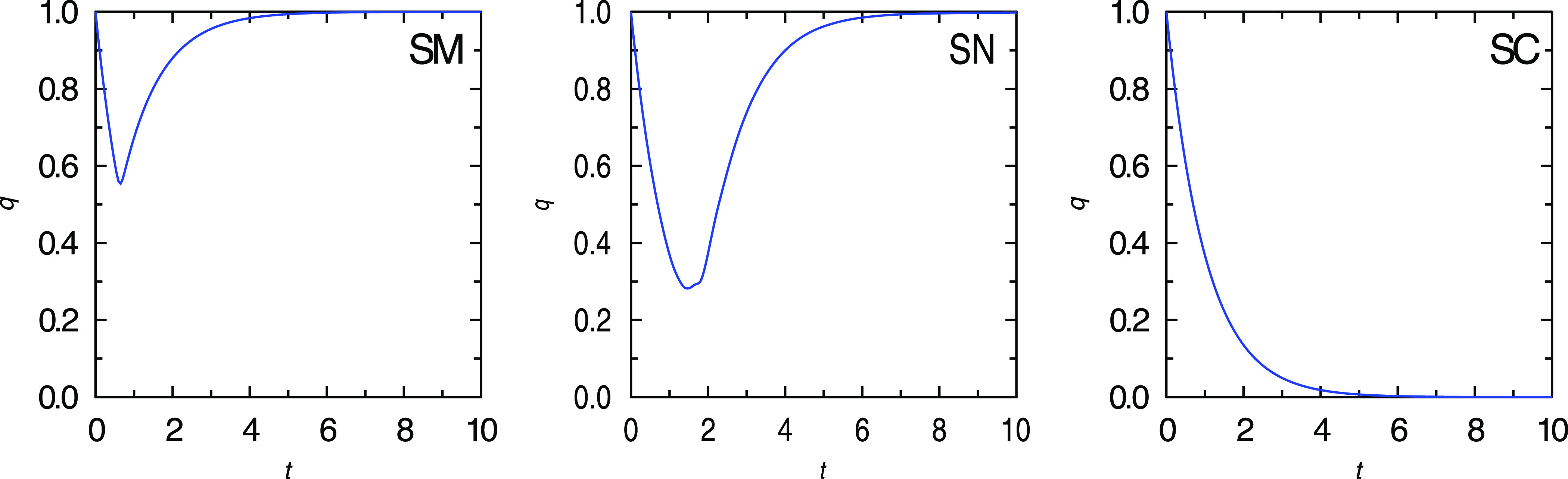
Temporal evolution of the empire’s activity obtained from numerical simulations of the three distinct cases: satellite mutualism (left), satellite neutralism (middle), and satellite competition (right).

*Interpretation:* *Competition of regions (provinces) for resources of the center diminishes empire stability. The mutualistic interaction between satellites enhances the rate of recovery of empire after crisis* (see Fig. 2).

In the neutral case $ES_{1,i}=0$, we should take into account the next dominating term $S_{2,i}$. If all coefficients are independent and identically distributed according to normal law $\text{Norm}(0,b^{2})$, then $ES_{2,i}=b^{2}\sum_{j=1}^{N}{\bar{w}}_{j}^{2}$. Since the second derivative $\sigma^{\prime \prime }(x)$ is negative for large x>0 and positive for large negative x, it means that a weak random satellite interaction diminishes the center activity if the center is active and that the interaction increases the center activity when the center is passive (q≈0).

As for strong interactions, this question is outside the scope of the paper, but if satellites interact strongly, then the centers are not capable to control them.

### C. Numerical simulations

In this section, we study how noise influences the stability of the empire. We apply the pure empire setting $C_{ij}=0$ given by the *divide and rule* principle. With respect to noise $\xi_{i}(t)$, we assume that it is white noise with zero mean and deviation μ. We will distinguish different cases when satellites are noised independently or in a correlated way. Survivability of empire is determined by its homeostasis domain,

Π={q∈R:q>δ}.

**FIG. 3.**
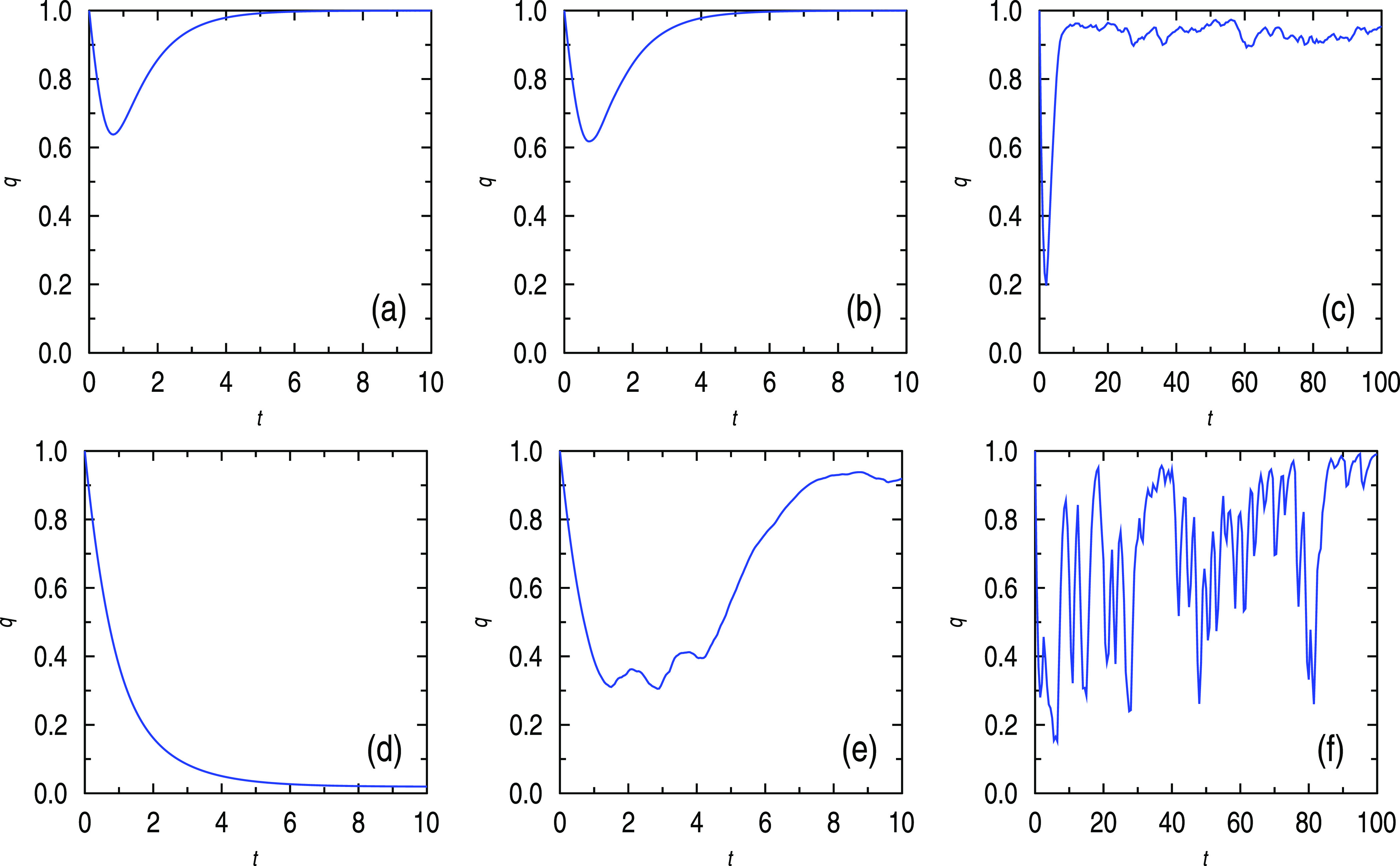
Temporal evolution of the empire’s activity obtained from numerical simulations of different cases: (a) small influence $h=h_{0}$, no white noise μ=0; (b) small influence $h=h_{0}$, small white noise $\mu =\mu_{0}$; (c) small influence $h=h_{0}$, big white noise $\mu =M\gg \mu_{0}$ (correlated/uncorrelated); (d) big influence h=H, small white noise $\mu =\mu_{0}$; (e) big influence, big white noise $\mu =M\gg \mu_{0}$ (uncorrelated); and (f) big influence h=H, big white noise μ=M (correlated).

The results of our numerical simulations are illustrated in Fig. 3 showing the temporal evolution of q for different parameter configurations.

Figure 3 has natural interpretations. External influence and noise average or the mean value of noise are the most critical terms for viability of empire for t→∞. Small noise provokes a crisis in empire, and then for time $t>T_{1}$, empire returns to a homeostasis domain (a)–(c). Alternatively, big influence implies a crash (d), but white noise could even prevent empire from fall (e). Notice that in the application domain, white noise can be related to concepts such as freedom on the one side, but also to corruption on the other side. Although corruption has a negative influence on the economy, it serves positively for viability because bureaucracy creates certain levels of resistance to external influence and propaganda. The simulations indicate that empires are rather stable with respect to an uncorrelated influence but becomes vulnerable when correlated action appears. Notice that big countries could suffer from correlated noise, not only because of synchronized attacks, but also because of being involved in a global market.

## VI. CONCLUSION

In this paper, centralized networks composed of multiple satellites arranged around a few dominating super-egoistic centers were studied. These structures that we called empires are organized using a divide and rule framework enforcing strong center–satellite interactions while keeping the pairwise interactions between the satellites sufficiently weak. The large *divide and rule* networks are stable under some conditions to center–satellite interactions. Theorem 1 states results on the existence of inertial manifolds in the dynamics of *divide and rule* networks. The existence of inertial dynamics is possible if the center mobility parameter κ is small enough.

Moreover, the stochastic stability of equilibrium states in centralized networks is studied. Under certain assumptions, it is shown that the probability to be in this state within a time T can be estimated by an interesting relation, which admits an analogy with physics, similar to Ref. 12. It can be connected with the energy of some system of particles occupying discrete states. The minimum of this energy corresponds to the maximal probability to stay in the steady state. If the noises acting on nodes are not correlated, then the form of the energy shows that we are dealing with a system of non-interacting particles. The energy minimum attains a state with a single center. In the opposite case, when the noises are strongly correlated, the network should have many nodes with the maximal possible connectivity.

Finally, our main conclusions are as follows:

1. Empires with n centers and $N_{s}\gg 1$ satellites are capable to generate all structurally stable dynamics of dimension n, for example, periodic or chaotic dynamics. The dynamics can be controlled by adjusting interaction forces between centers and satellites appropriately (Theorem 2).
2. If noises acting on nodes in a network with a few organizing centers are positively correlated, then the optimal state of the networks attains at maximal possible center connectivity. In particular, the structure with a single center sharing the maximum of possible links is most stable.
  However, if we have even a weak negative correlation, then the optimal (the most stable) structure of the networks is absolutely another. Here, the connectivity of each center is bounded and depends on the correlation level.
3. According to Theorem 1, the control by centers is possible only when the centers evolve slowly. Except for short periods when the state approaches to inertial manifold, the development of such structures is very slow. Therefore, we can say that with increasing size, these structures eventually end up in “the control trap.” As a result, they are not capable to evolve.

The idea that the control trap is a main obstacle for development is consistent, as we think, with contemporary economical ideas. An important conclusion of the contemporary economic–social models (see Refs. 44 and 45) is that economic growth may be accompanied by conflict interests of various economic agents. Since the development of new products leads to loss of monopoly rent by firms already existing on the market, the latter will have an incentive to impede technological progress. If owners of existing firms have significant political weight and the ability to influence economic policy, then to protect them, interests will lead to a slowdown in economic growth. In terms of the model, this means multiple decrease in research technology productivity as the introduction of new technologies becomes more costly; then, the structural stability becomes an obstacle to growth.

## Data Availability

The data that support the findings of this study are available from the corresponding author upon reasonable request.
